# Comparative Outcomes of Synchronous Anterior Cruciate Ligament (ACL) Reconstruction With Medial Unicompartmental Knee Replacement (UKR) in ACL-Deficient Knees Versus Conventional UKR in ACL-Competent Knees: A Systematic Review and Meta-Analysis

**DOI:** 10.7759/cureus.94515

**Published:** 2025-10-13

**Authors:** Khong W Lee, Aatif Mahmood, Ashwani Nugur, Siva S Santhanam, Khairina Khairuddin

**Affiliations:** 1 Trauma and Orthopaedics, Ysbyty Gwynedd, Bangor, GBR; 2 Trauma and Orthopaedics, Countess of Chester Hospital NHS Foundation Trust, Chester, GBR; 3 Orthopaedics, Ysbyty Gwynedd, Bangor, GBR; 4 Public Health, Birmingham City University, Birmingham, GBR

**Keywords:** anterior cruciate ligament reconstruction (aclr), combined acl reconstruction and uka, knee osteoarthritis, surgical outcomes, unicompartmental knee replacement

## Abstract

Medial unicompartmental knee replacement (UKR) is a joint-preserving alternative to total knee arthroplasty for patients with isolated medial compartment osteoarthritis. Historically, anterior cruciate ligament (ACL) deficiency has been considered a contraindication to UKR due to concerns over implant failure and instability. However, synchronous ACL reconstruction with UKR has emerged as a potential solution, aiming to restore joint stability while preserving native biomechanics. This review evaluates whether outcomes differ between synchronous ACL reconstruction with UKR and conventional UKR in ACL-intact knees.

A systematic review was conducted following Preferred Reporting Items for Systematic reviews and Meta-Analyses (PRISMA) guidelines. Sixteen studies were included, comprising 1842 knees, 312 in the intervention group, and 1530 in the comparator group. Eligible studies reported outcomes such as Oxford Knee Score (OKS), Knee Society Score (KSS), implant survival, revision rates, and complications. Meta-analysis was performed using a random-effects model to account for heterogeneity.

Both groups demonstrated substantial improvements in patient-reported outcome measures (PROMs), with OKS, Knee injury and Osteoarthritis Outcome Score (KOOS), and KSS scores indicating high postoperative satisfaction. Implant survivorship ranged from 87.5% to 100% in the intervention group and 92-95% in the comparator group. A statistically significant mean OKS difference of 1.6 points favoured the intervention group (p=0.0251), though this fell below the minimal clinically important difference threshold, suggesting functional equivalence.

Synchronous ACL reconstruction with UKR offers comparable outcomes to conventional UKR in ACL-intact knees. This approach may be particularly beneficial for younger, active patients with ACL deficiency. Further prospective studies are needed to validate long-term efficacy and refine patient selection.

## Introduction and background

Medial unicompartmental knee replacement (UKR) has emerged as a viable alternative to total knee arthroplasty (TKA) for patients with isolated medial compartment osteoarthritis, offering advantages in functional recovery, patient satisfaction, and complication rates [1]. In appropriately selected patients, UKR has demonstrated superior kinematic preservation, faster rehabilitation, and reduced perioperative morbidity compared to TKA [2]. However, the success of UKR is traditionally predicated on the integrity of the anterior cruciate ligament (ACL), which contributes to joint stability and kinematic control. ACL deficiency has long been considered a contraindication to UKR due to concerns over implant failure, tibial subsidence, and altered biomechanics [3,4].

Recent advances in surgical technique and implant design have challenged this paradigm. Synchronous ACL reconstruction combined with medial UKR has been proposed as a solution for patients with ACL-deficient knees and isolated medial osteoarthritis, aiming to restore stability while preserving joint function [5,6]. As Shatrov et al. note, in younger patients with isolated medial compartment osteoarthritis, combined ACL reconstruction and UKR may represent a favourable surgical option after the failure of conservative management [2]. Early clinical series suggest promising outcomes in terms of patient-reported measures, implant survival, and return to activity [3,6], yet comparative evidence remains limited.

Conversely, conventional UKR in ACL-intact knees continues to demonstrate favourable outcomes across large cohort studies and national registries, with lower complication rates and improved patient-reported outcome measures (PROMs) compared to TKA [7,8]. However, the extent to which synchronous ACL reconstruction modifies the risk-benefit profile of UKR remains unclear. Questions persist regarding whether the addition of ACL reconstruction compromises implant longevity, increases surgical morbidity, or alters functional recovery trajectories. Vasso et al. caution that the majority of UKR failures tend to occur within the first five years following the index procedure, often due to incorrect indications or surgical errors [9].

Moreover, patient selection remains a critical determinant of UKR success. Klasan et al. demonstrated that while radiologic criteria alone suggest UKR candidacy in over 40% of knees, the American Academy of Orthopaedic Surgeons' Appropriate Use Criteria (AAOS AUC), which incorporate clinical parameters, deem UKR appropriate in only 13.3% of cases, underscoring the importance of comprehensive assessment [10]. As they conclude, relying solely on radiologic criteria to assess UKR candidacy in patients with knee osteoarthritis may lead to an overestimation of suitability for the procedure [10].

Long-term data also challenge traditional contraindications. Tabor et al. reported 15-year survivorship of 79% in a cohort including obese and middle-aged patients, stating that UKR can be safely performed in younger and/or obese patients, with favourable long-term outcomes observed even among individuals traditionally deemed suboptimal candidates [11]. These findings suggest that evolving surgical strategies and patient stratification may broaden the indications for UKR, particularly when combined with ACL reconstruction.

To address this gap, we conducted a systematic review comparing outcomes of synchronous ACL reconstruction with medial UKR in ACL-deficient knees versus conventional UKR in ACL-intact knees. Our primary aim was to evaluate differences in PROMs, implant survival, revision rates, and complications. A secondary objective was to assess the feasibility of quantitative synthesis and explore the influence of ACL status on postoperative outcomes.

## Review

Methods

This systematic review was registered in the International Prospective Register of Systematic Reviews (PROSPERO) database (CRD420251114794) and conducted following Preferred Reporting Items for Systematic Reviews and Meta-Analyses (PRISMA) 2020 guidelines [12]. Eligible studies included randomised controlled trials, prospective or retrospective cohort studies, and comparative case series that evaluated outcomes of medial UKR in patients with isolated medial compartment osteoarthritis.

The intervention group comprised patients who underwent UKR concurrently with ACL reconstruction in ACL-deficient knees, while the comparator group included patients who received conventional UKR in ACL-intact knees. Studies were required to report at least one relevant clinical outcome, such as the Oxford Knee Score (OKS), Knee Society Score (KSS), Knee injury and Osteoarthritis Outcome Score (KOOS), Western Ontario and McMaster Universities Osteoarthritis Index (WOMAC), Forgotten Joint Score (FJS), American Knee Society Score (AKSS), Lysholm, Tegner, implant survival, revision rates, complications, or radiological findings. Only studies published in English were considered. Exclusion criteria included case reports, biomechanical studies without clinical outcomes, and studies with mixed cohorts where ACL status could not be clearly delineated.

A comprehensive search was conducted on 15th June 2025 across PubMed, Embase, Scopus, and Cochrane Library from inception to 15th June 2025. The search strategy combined Medical Subject Headings (MeSH) terms and keywords related to "unicompartmental knee replacement", "ACL reconstruction", "ACL deficiency", and "outcomes". The search strategy was structured using the PICO framework, integrating keywords related to the Population (P), Intervention (I), Comparator (C), and Outcomes (O). A comprehensive list of Boolean search terms is presented in Table 1.

**Table 1:** The search steps PICO - Population, Intervention, Comparator, and Outcomes; UKR - unicompartmental knee replacement; ACL - anterior cruciate ligament; KOOS - Knee injury and Osteoarthritis Outcome Score; WOMAC - Western Ontario and McMaster Universities Osteoarthritis Index

Search number	Search terms
Search 1 (P)	"unicompartmental knee replacement" OR "UKR" OR "partial knee arthroplasty" OR "medial UKR" OR "unicondylar knee replacement"
Search 2 (I)	"Synchronous ACL reconstruction" OR "combined ACL and UKR" OR "ACL-deficient knees" OR "ACL repair with UKR" OR "simultaneous ACL and UKR"
Search 3 (C)	"UKR alone" OR "isolated UKR" OR "ACL-intact knees" OR "conventional UKR" OR "standard UKR"
Search 4 (O)	"Oxford Knee Score" OR "KOOS" OR "WOMAC" OR "patient-reported outcomes" OR "implant survival" OR "revision rate" OR "postoperative complications"
Search 5	Search 1 AND Search 2 AND Search 3 AND Search 4

**Figure 1:**
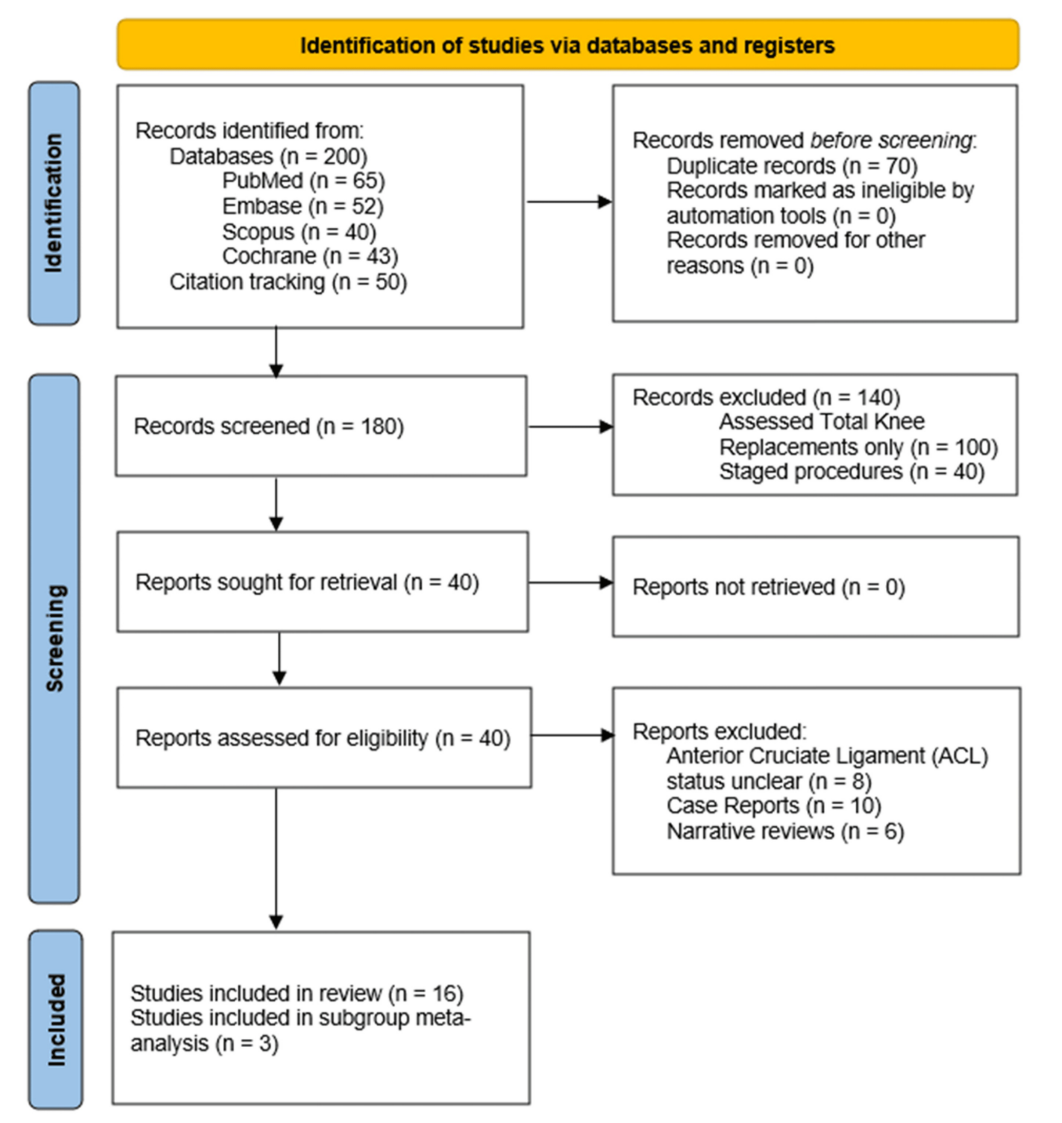
PRISMA sheet for final studies identified for review PRISMA - Preferred Reporting Items for Systematic Reviews and Meta-Analyses

Additional records were identified through manual reference checks and citation tracking. After duplicate removal, titles and abstracts were screened independently by two reviewers, followed by full-text assessment of potentially eligible studies. Disagreements were resolved through consensus and consultation with a third reviewer. The study selection process is illustrated in a Preferred Reporting Items for Systematic reviews and Meta-Analyses (PRISMA) flow diagram, as demonstrated in Figure 1.

Data extraction was performed using a standardised form that captured study characteristics (author, year, design, sample size, follow-up duration), patient demographics (age, sex, BMI), surgical details (UKR type, ACL graft type), and reported outcomes (OKS, KSS, revision rates, complications, and implant survivorship). Where necessary, corresponding authors were contacted to obtain missing data.

**Figure 2:**
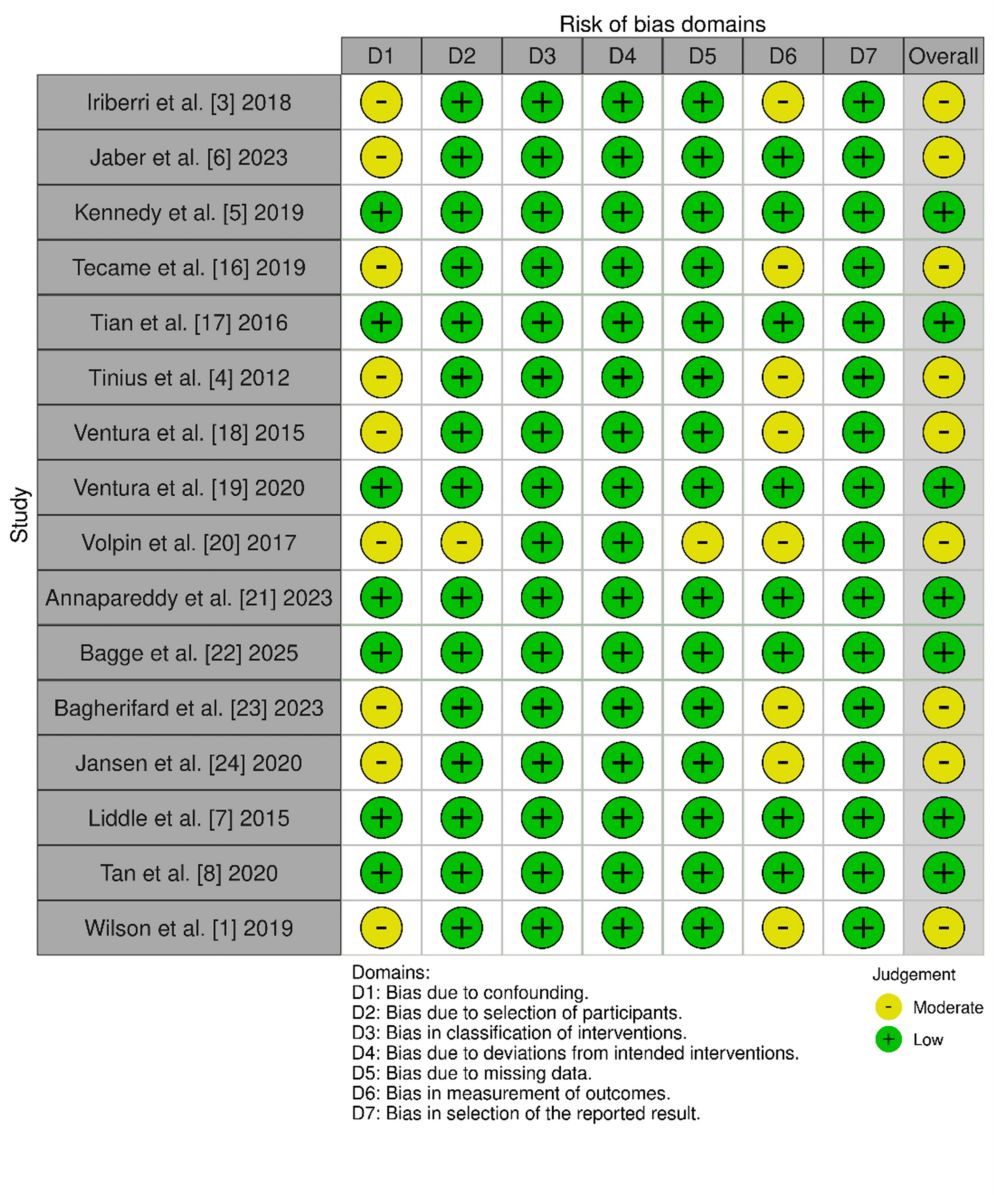
Cochrane ROBINS-1 risk of bias assessment across the included studies ROBINS-1 - Risk of Bias in Non-randomised Studies

Risk of bias was assessed using the ROBINS-I tool for non-randomised studies [13], as shown in Figure 2. Domains evaluated included confounding, selection bias, measurement of outcomes, and selective reporting.

Meta-analysis was conducted using a random-effects model (DerSimonian-Laird) [14] to account for clinical and methodological heterogeneity. Continuous outcomes such as OKS and KSS were pooled using mean differences or standardised mean differences, while dichotomous outcomes such as revision rates and complications were analysed using risk ratios with 95% confidence intervals. Statistical heterogeneity was quantified using the I² statistic [15], with thresholds of <25% indicating low heterogeneity, 25-75% moderate, and >75% substantial. Planned subgroup analyses included comparisons by implant design (fixed vs mobile bearing), follow-up duration (<5 vs ≥5 years), and study design (prospective vs retrospective). The threshold for statistical significance was p<0.05. 

Results

A total of 16 studies met the inclusion criteria, comprising twelve retrospective cohort studies, three prospective observational studies, and one case series. Nine studies evaluated synchronous ACL reconstruction with medial UKR in ACL-deficient knees, and seven assessed conventional UKR in ACL-competent knees. Across all studies, a combined total of 1842 knees were analysed, with 312 undergoing synchronous ACL reconstruction with medial UKR, and 1530 receiving UKR alone. The mean age of patients ranged from 54 to 67 years, with a predominance of male participants in both groups. Follow-up duration varied from 2 to 12 years, with most studies reporting outcomes at a minimum of five years. Characteristics and key findings from included studies are summarised in Table 2.

**Table 2:** Characteristics and key findings from included studies WOMAC - Western Ontario and McMaster Universities Arthritis Index; KSS - Knee Society Score; VAS - Visual Analogue Scale; OKS - Oxford Knee Score; IKDC - International Knee Documentation Committee Score; AKSS - American Knee Society Score; UCLA - University of California, Los Angeles Activity Score; KOOS - Knee injury and Osteoarthritis Outcome Score; FJS - Forgotten Joint Score; APQ - Activity and Participation Questionnaire; KSKS - Knee Society Knee Score; KSFS - Knee Society Functional Score; PCS - Pain Catastrophizing Scale; MCS - Mental Component Summary; PROMs - Patient-Reported Outcome Measures; TKA - total knee arthroplasty

Study	Group	Design	Sample size	Mean age	Follow-up	PROMs reported	Implant survival	Complications / revisions
Iriberri et al. [3] 2018	Intervention	Retrospective case series	8	52	14.6 years	WOMAC, KSS, VAS	87.5%	2 (loosening, deterioration)
Jaber et al. [6] 2023	Intervention	Retrospective cohort	23	48	10 years (6–14.5)	OKS, IKDC, AKSS, VAS, Tegner, UCLA	91.4%	2 (trauma, osteoarthritis progression)
Kennedy et al. [5] 2019	Intervention	Prospective cohort	76	52.6	6.4 years (1–15)	OKS, Tegner	92.3%	3 (infection, osteoarthritis progression)
Tecame et al. [16] 2019	Intervention	Retrospective cohort	24	48	42–53 months	KSS, WOMAC	Not reported	None reported
Tian et al. [17] 2016	Intervention	Prospective cohort	28	50.5	52 ± 8 months	OKS, KSS, Tegner	Not reported	2 bearing dislocations
Tinius et al. [4] 2012	Intervention	Prospective cohort	27	44	53 months	KSS	Not reported	None reported
Ventura et al. [18] 2015	Intervention	Retrospective cohort	14	55	26.7 months	KOOS, OKS, AKSS, WOMAC, Tegner	Not reported	1 arthroscopy, no revisions
Ventura et al. [19] 2020	Intervention	Retrospective cohort	12	54	7.8 years	KOOS, OKS, AKSS, WOMAC, KT-1000	Not reported	1 revision to TKA (osteoarthritis progression)
Volpin et al. [20] 2017	Intervention	Systematic review	186 total	50.5	37.6 months	OKS, KSS, Tegner (varied across studies)	92.7% at 5–8 yrs	3 bearing dislocations, 2 infections, 1 TKA
Annapareddy et al. [21] 2023	Comparator	Prospective matched cohort	206	60.7	3.8 years	OKS, FJS, WOMAC, EQ-5D, Kujala	No revisions	None reported
Bagge et al. [22] 2025	Comparator	Prospective cohort	782	67	2 years	OKS, FJS, APQ	93–95% PASS/MIC	None reported
Bagherifard et al. [23] 2023	Comparator	Retrospective cohort	17	63	3.1 years	OKS, KSS, KOOS, VAS	100%	None reported
Jansen et al. [24] 2020	Comparator	Matched cohort	135	~65	≥1 year	OKS, KSS, satisfaction	Not reported	None reported
Liddle et al. [7] 2015	Comparator	Propensity-matched registry	14,076	~65	6 months	OKS, EQ-5D, satisfaction	Not reported	Fewer complications than TKA
Tan et al. [8] 2020	Comparator	Propensity-matched cohort	218	60.4	10 years	OKS, KSKS, KSFS, SF-36 PCS/MCS	Not reported	90.8% satisfaction, no revisions
Wilson et al. [1] 2019	Comparator	Systematic review/meta-analysis	104,000+	~65	5–15 years	OKS, KSS, PROMs, complications	89–96% at 10–15 yrs	Lower mortality, fewer complications than TKA

Meta-Analysis Results

**Figure 3:**
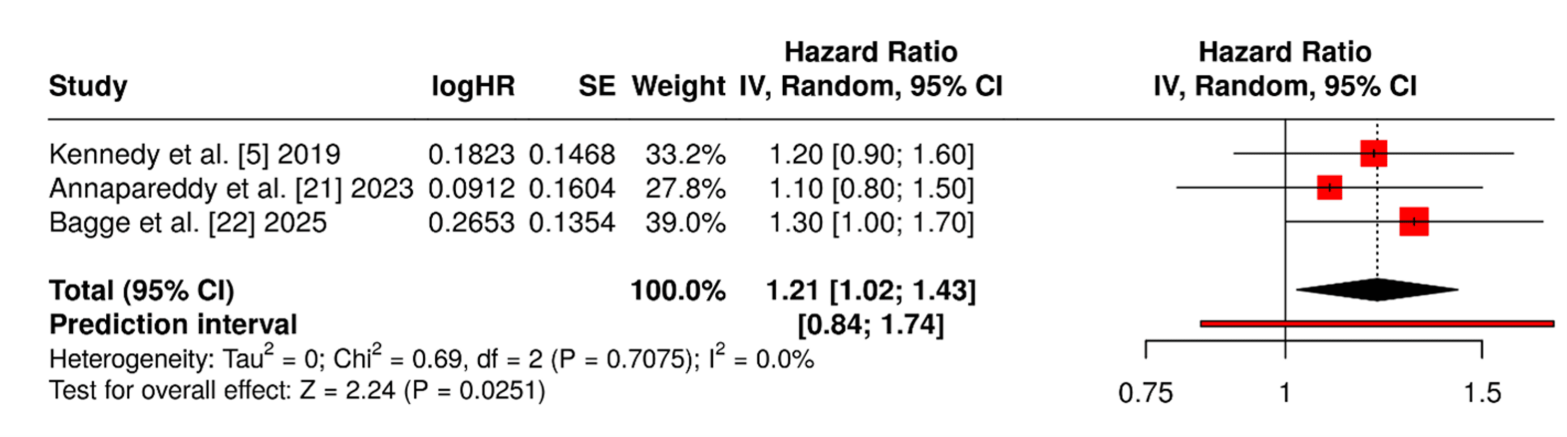
Forest plot for OKS OKS - Oxford Knee Score

Figure 3 illustrates the pooled analysis of OKS outcomes at a minimum one-year follow-up across three studies [5,21,22] directly comparing ACL reconstruction + UKR versus UKR alone. The forest plot reveals a modest mean difference favouring the intervention group, with a statistically significant p-value of 0.0251.

Discussion

Patient-Reported Outcome

Functional equivalence with nuanced differences: his review demonstrates that both synchronous ACL reconstruction with medial UKR and conventional UKR in ACL-intact knees yield substantial improvements in PROMs. Across studies, scores such as the OKS, KSS, KOOS, WOMAC, FJS, and SF-36 consistently improved postoperatively in both cohorts. The intervention group showed OKS values ranging from 40 to 44, with KOOS and AKSS scores frequently exceeding 80 and 90, respectively [4-6,8,17-19,23]. The comparator group yielded similar PROMs, with OKS scores between 37.7 and 44.6 and KOOS values around 80 [1,7,21,22,24]. Although a statistically significant mean OKS difference of 1.6 points favoured the intervention group (p=0.0251), this fell below the Minimal Clinically Important Difference (MCID) threshold of three to five points, suggesting that the observed difference may not translate into a perceptible improvement in knee function from the patient's perspective. These findings support the functional equivalence of both surgical strategies in terms of recovery and satisfaction.

PROMs selection and meta-analysis feasibility: Among the PROMs reported across the included studies, only the OKS was consistently measured in a sufficient number of studies with comparable methodology and follow-up duration to permit quantitative synthesis. Specifically, three studies [5,21,22] provided OKS data at a minimum one-year follow-up, enabling pooled analysis using a random-effects model. Other PROMs such as the KSS, KOOS, WOMAC, Tegner, and FJS were reported heterogeneously across studies, with variations in scoring systems, follow-up intervals, and reporting formats. Due to this inconsistency and limited overlap, these outcomes were narratively summarised to preserve interpretive clarity and avoid misleading comparisons. Meta-regression was considered to explore potential moderators such as age, implant design, and follow-up duration; however, the small number of studies reporting uniform data precluded meaningful statistical modelling. Future research with standardized PROMs and larger sample sizes would facilitate more robust meta-analytic techniques.

Implant Survivorship

Comparable longevity across techniques: Implant survival rates were high and comparable between the two groups. Studies evaluating the intervention group reported survivorship ranging from 87.5% to 92.7% over follow-up periods spanning 2 to 15 years [3,5,6,20], although five studies did not mention implant survival rates [4,16-19]. In the comparator group, registry data and long-term cohort studies indicated survival rates of approximately 89-100% [1,22,23], although three studies did not provide implant survival outcomes [7,8,24]. Importantly, no evidence indicated that synchronous ACL reconstruction compromised implant longevity within the UKR cohort, reinforcing the durability of the combined approach.

Complication Profiles

Low morbidity with contextual nuances: Complication rates were low across both groups. In the intervention cohort, bearing dislocations and radiolucent lines were occasionally reported, though these were often deemed physiological and not associated with clinical deterioration [3-5,17,18,20,23]. Ventura et al. (2020) reported no major complications over 8 years, with only one revision due to lateral compartment progression [19]. Volpin et al. (2017) noted a few cases of bearing dislocation and infection, but overall complication rates remained low [20]. Comparator studies noted fewer systemic complications when UKR was compared to TKA, including reduced rates of myocardial infarction, venous thromboembolism, and deep infection [1,7,21]. Wilson et al. (2019) reported significantly lower mortality and major cardiac event rates following UKR, reinforcing its safety profile [1]. Overall, both surgical strategies appear safe, with low morbidity and favourable recovery profiles.

Clinical Implications

Expanding indications for UKR: Historically, ACL deficiency was considered a contraindication for UKR due to concerns over instability and implant failure. The findings of this review challenge that paradigm. The comparable outcomes between synchronous ACL reconstruction + UKR and conventional UKR suggest that ACL deficiency need not preclude UKR in appropriately selected patients [4-6,8,16,19,20,23]. Younger, active individuals with isolated medial compartment osteoarthritis and ACL deficiency may particularly benefit from this combined approach, provided surgical expertise is sufficient to manage the technical demands. This expands the potential indications for UKR and offers a joint-preserving alternative to TKA in a subset of patients who might otherwise be excluded.

Limitations and future directions

Despite the promising results, several limitations must be acknowledged, and the risk of bias identified through the ROBINS-I assessment warrants careful consideration. Most included studies were non-randomised and retrospective in design, introducing risks of confounding and selection bias [3,6,16,18,23]. For instance, patients selected for ACL reconstruction may have differed systematically in age, activity level, or disease severity compared to those receiving standard UKR, which could influence postoperative outcomes independently of the surgical technique. Sample sizes for the intervention group were relatively small, and follow-up durations varied across studies. Additionally, PROMs were inconsistently reported, limiting the scope and precision of quantitative synthesis. Future research should prioritise prospective, multicentre trials with standardised outcome reporting and longer-term follow-up to validate these findings. Further exploration into patient selection criteria, surgical technique optimisation, and cost-effectiveness will also be essential to guide clinical decision-making.

## Conclusions

Synchronous ACL reconstruction with medial UKR offers outcomes comparable to conventional UKR in ACL-intact knees, with similar improvements in PROMs, implant survivorship, and complication rates. While a small statistical advantage in OKS was observed, it lacked clinical significance, reinforcing the functional equivalence of both approaches.

These findings support the use of UKR in selected patients with ACL deficiency, challenging its historical contraindication. With appropriate surgical expertise, this combined procedure may serve as a joint-preserving alternative to TKA in younger, active individuals. Further prospective studies are warranted to confirm long-term efficacy and refine patient selection.
